# Phospho-ERK1/2 levels in cancer cell nuclei predict responsiveness to radiochemotherapy of rectal adenocarcinoma

**DOI:** 10.18632/oncotarget.5761

**Published:** 2015-09-21

**Authors:** Susanne Holck, Hans Jørgen Nielsen, Niels Pedersen, Lars-Inge Larsson

**Affiliations:** ^1^ Department of Pathology, Copenhagen University Hospital, Hvidovre, Copenhagen, Denmark; ^2^ Department of Surgical Gastroenterology, Copenhagen University Hospital, Hvidovre, Copenhagen, Denmark; ^3^ Clinical Research Centre, Copenhagen University Hospital, Hvidovre, Copenhagen, Denmark

**Keywords:** ERK, radiochemotherapy, rectal carcinoma, immunohistochemistry, phosphorylation

## Abstract

Locally advanced rectal adenocarcinoma is treated with radiochemotherapy (RCT) before surgery. The response to RCT is heterogeneous and consensus regarding reliable predictors is lacking. Since the ERK pathway is implicated in radioprotection, we examined pretreatment biopsies from 52 patients by immunohistochemistry for phosphorylated ERK (pERK). Immunostaining for pERK was considerably enhanced by use of alkaline demasking. Nuclear staining occurred in both cancer cells and stromal cells. Blind-coded sections were scored by 2 independent investigators. In patients showing no residual tumor after RCT (TRG1), staining for pERK in cancer, but not stromal, cell nuclei was significantly weaker than in patients showing a poor RCT response (TRG1 vs TRG4: *p* = 0.0001). Nuclear staining for pERK predicted poor responders, as illustrated by receiver operating characteristic curves with an area under curve of 0.86 (*p* = 0.0007) and also predicted downstaging (area under curve: 0.76; *p* = 0.01). A number of controls documented the specificity of the optimized staining method and results were confirmed with another pERK antibody. Thus, staining for pERK in cancer cell nuclei can predict the response to RCT and may help spare poor responders this treatment. These results also raise the question whether inhibitors of ERK activation may serve as response modifiers of RCT.

## INTRODUCTION

Rectal adenocarcinoma is associated with considerable mortality and morbidity. Treatment has been improved by optimized surgical methods and by preoperative radio- and chemotherapy (RCT) of patients with locally advanced disease. Following RCT, complete tumor regression (pathological complete response) is obtained in around 15 % of patients, whereas others show no or partial responses [1, 2]. Complete tumor regression has been correlated to improved long-term outcome in several, but not all, studies [see references 1, 2 and references therein]. It is important to find markers that can predict responses to RCT in order to spare poor responders this treatment and to detect potential response-modifying pathways. Although several promising markers have been unveiled there is no consensus regarding such markers [3-5].

We considered that activated, dually phosphorylated extracellular signal-regulated kinase 1 and 2 (henceforth referred to as pERK) might constitute such a marker. Phosphorylation of ERK1/2 occurs in response to activation of growth factor receptors with subsequent activation of the RAS-RAF-MEK-ERK signaling cascade [6, 7]. Increased activation of the EGF receptor or activating mutations in RAS or B-RAF characterizes a substantial proportion of colorectal carcinomas [6]. pERK phosphorylates a multitude of cytoplasmic and nuclear proteins and increases transcription of the cyclin D1 gene and cell cycle progression [6, 7]. Additionally, pERK has antiapoptotic effects [5]. Recent data show that pERK stimulates survivin expression, which may contribute to its antiapoptotic role [8, 9]. ERK phosphorylation affords protection against ionizing radiation in cultured tumor cells and inhibitors of its upstream kinases, MEK1 and 2, restores radiosensitivity [10, 11]. Such inhibitors are in clinical trials [12] but their potential for radiation response modification remains unexplored. Additionally, staining for pERK has been correlated to poor responsiveness to radiotherapy in glioblastomas [13]. Conceivably, the radioprotective effects of pERK may reflect its antiapoptotic effects. Using an optimized immunohistochemical method we therefore investigated whether levels of pERK in cancer cells or stromal cells could predict the response to preoperative RCT in rectal adenocarcinoma.

## RESULTS

**Figure 1:**
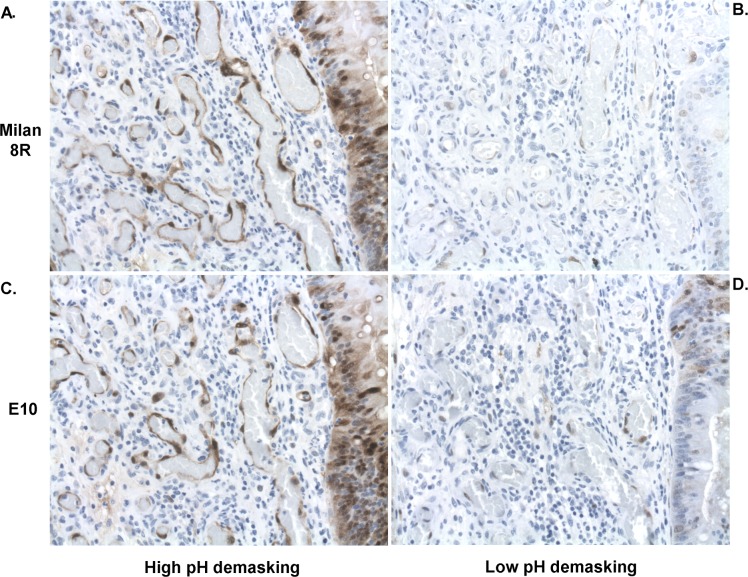
Adjacent sections of pretreatment biopsies demonstrating effects of high **A.**, **C.** and low **B.**, **D.** pH demasking on staining with the Milan8R **A.**, **B.** and E10 **C.**, **D.** pERK antibodies. Note vastly more intense staining with both antibodies after high pH demasking. Staining occurs in both nuclei and cytoplasm but is most intense in nuclei. Both endothelial cells and tumor cells (right) are stained following appropriate demasking.

**Figure 2:**
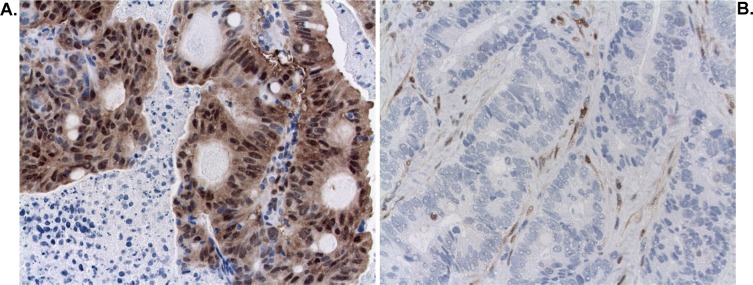
Staining for pERK (Milan8R antibody) in cancer and stromal cell nuclei **A.** Pretreatment biopsy showing strong staining for pERK in cancer cell nuclei and less staining in stromal cell nuclei (TRG4). **B.** Pretreatment biopsy showing strong staining for pERK in stromal cell nuclei and no staining of cancer cell nuclei (TRG1).

**Figure 3:**
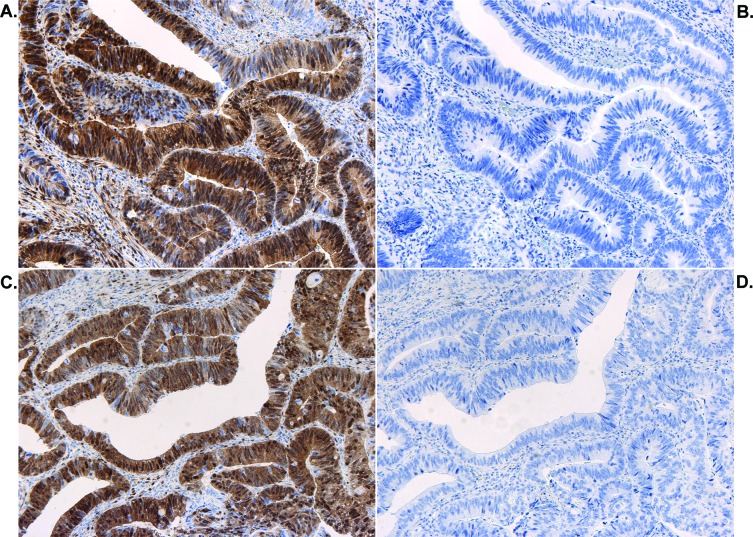
Controls for pERK staining Adjacent sections stained with the pERK (Milan8R) antibody **A.** or with control IgG1 monoclonal antibody **B.** and adjacent sections pretreated with buffer alone **C.** or with lambda protein phosphatase **D.** and then stained with the pERK (Milan8R) antibody.

A training set of biopsies were initially stained for pERK using 2 different monoclonal antibodies (Milan8R and E10) by our standard method involving high pH demasking. Final dilutions were determined using this method (Figure 1A and 1C). Demasking at low pH, which is recommended by the antibody vendors and which represents the standard method of demasking for pERK staining, yielded much inferior results at the same antibody dilutions (Figure 1B and 1D). Both antibodies detected staining of stromal cells and cancer cells. Variable proportions of stromal and cancer cells were stained in individual specimens but all specimens showed a uniform staining pattern throughout the blocks with no evidence of a gradient. Staining was strongest in nuclei and weaker in the cytoplasm (Figures 1 and 2). Positive stromal cells included endothelial cells (Figure 1) and myofibroblast-like cells (Figures 2 and 3A). Of the two antibodies tested, the Milan8R antibody produced more crisp staining than the E10 antibody and had the advantage that the IgG1 concentration was indicated so that matching concentrations of irrelevant (Aspergillus nigricans glucose oxidase) IgG1 could be used for controls. Such controls, as well as dephosphorylation prior to immunostaining, were consistently negative (Figure 3).

Pretreatment biopsies from patients selected for RCT treatment at the Copenhagen University Hospital at Hvidovre during 2006-2013 (Table 1) were subsequently examined. All consecutive patients were included. However, biopsies from 7 patients that had been examined by private practicing pathologists were not made available to us. The material examined comprised 52 patients (24 females) with a median age of 62.5 years (range 40-86 years). The responses to RCT were assessed on surgical resection specimens by the method of Mandard et al. [14, 15] and were confirmed by an experienced GI pathologist (SH). According to this scale, TRG1 implies total tumor regression and TRG4 implies a poor response to therapy. TRG1 was obtained in 17%, TRG2 in 31%, TRG3 in 35% and TRG4 in 17% of the patients. No patients with total lack of response to RCT (TRG5) were retrieved from the records. Downstaging was assessed by subtracting pathological T stage (pT) from clinical T stage (cT, as assessed by magnetic resonance imaging). These data were available for 46 of the 52 patients. Eight of the 9 patients with TRG 1 showed major downstaging (cT-pT≥2, pN0) (cT and cN was unavailable for one) whereas only one of the remaining patients (TRG2) showed major downstaging (Table 1).

**Table 1:** Patient characteristics

	TRG1	TRG2	TRG3	TRG4
pERK1/2 median (range)^a^	1.0 (0.0-2.0)^b^	1.0 (0.0-4.0)^c^	2.0 (0.0-6.0)^d^	4.0 (2.0-6.0)^e^
n (female:male)	9 (5f:4m)	16 (5f:11m)	18 (11f:7m)	9 (3f:6m)
age median (range)	63.0 (52-81) y	63.5 (46-81) y	62.5 (46-75) y	54.0 (40-86) y
radiotherapy^f^	9/9	16/16	18/18	7/9
pT0^g^	9/9	0/16	0/18	0/9
pT1^g^	0/9	2/16	0/18	1/9
pT2^g^	0/9	5/16	6/18	2/9
pT3^g^	0/9	7/16	9/18	6/9
pT4^g^	0/9	1/16	3/18	0/9
pTX^g^	0/9	1/16	0/18	0/9
pN0^h^	9/9	13/16	14/18	6/9
pN1^h^	0/9	3/16	2/18	2/9
pN2^h^	0/9	0/16	2/18	1/9
vascular invasion	0/9	0/16	1/18	0/9
neural invasion	0/9	2/16	2/18	1/9
differentiation^i^	8 mod; 1 p	13 mod; 3 p	1 h; 16 mod; 1 p	7 mod; 2 p
Tumor location^j^	3 mid; 6 low	1 high; 5 mid; 10 low	1 high; 7 mid; 10 low	4 mid; 5 low
Downstaging (cT-pT)^k^	3 (2-4)	0.3 (0-2)	0.0 (0-1)	0.0 (-1-1)

a denotes staining for pERK1/2 in cancer cell nuclei;

b significantly different from TRG3 (Mann-Whitney; p=0.03) and TRG4 (p=0.0001);

c significantly different from TRG4 (p=0.002);

d significantly different from TRG4 (p=0.02);

e significantly different from TRG1-3, also when two patients not receiving radiotherapy were excluded (p=0.001);

f 48-60 Gy over 25-27 days;

g denotes pathological tumor stage; pTX denotes a case of uncertain staging

h denotes pathological nodal stage;

i h denotes high; mod denotes moderate and p denotes poor or mucinous differentiation;

j low denotes 0-5 cm; mid: 5-10 cm and high: 10-15 cm from the anorectal junction;

k denotes the difference between clinical stage (cT) and pT given as the median and ranges.

Immunohistochemically stained sections were blind-coded and, following an initial survey, staining intensities of tumor and stromal cell nuclei were graded as nil (0), moderate (1) or strong (2) and numbers of stained cancer and stromal cell nuclei were graded as 0% (0), below 10% (1), 10-60% (2) and above 60 % (3). The intensity scores were multiplied with the number scores before statistical analyses. Scorings were undertaken by two independent investigators (SH and LIL), who were blinded to the clinical outcome. There was good consensus between the individual scores (kappa = 0.74) and final results were computed from averages of scores from both investigators.

**Figure 4:**
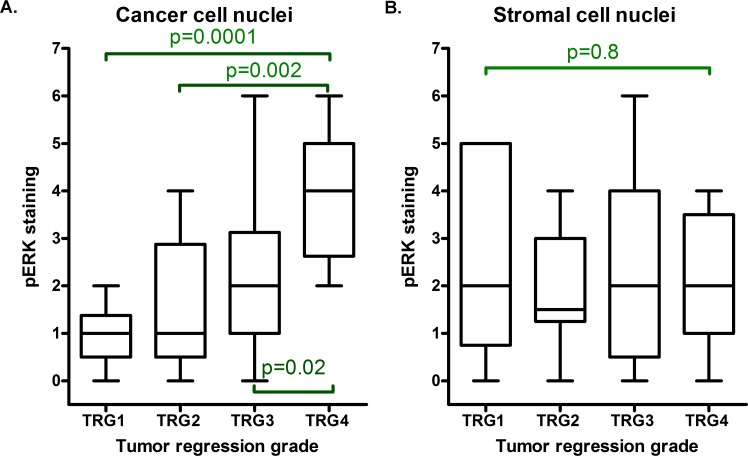
Box and whiskers plot demonstrating results of blind scorings of cancer and stromal cell nuclear staining with the pERK (Milan8R) antibody Averages of scorings from two observers are presented. Note that cancer cell nuclear staining **A.**, but not stromal cell nuclear staining **B.**, increases with the tumor regression grade (TRG1 identifies total tumor regression). Horizontal lines identify medians, boxes identify interquartile ranges and whiskers identify total ranges of scorings. The p values indicated in the figure refer to Mann-Whitney U tests of differences between individual groups. A Kruskal-Wallis test of all groups returns *p* = 0.001 for cancer cell nuclear staining and *p* = 0.990 for stromal cell nuclear staining.

The immunohistochemical scores (Milan8R antibody) revealed that there was significantly stronger staining of cancer cell nuclei in TRG4 tumors than in TRG1-3 tumors (Mann-Whitney test: *p* = 0.0007) and the difference between TRG1 and TRG4 tumors was significant at the *p* = 0.0001 level (Figure 4A and Table 1). In contrast, there was no relationship between TRG and staining of stromal cell nuclei (TRG1 vs. TRG4, *p* = 0.8; Figure 4B). Thus, stromal cells, which occurred closely intermingled with cancer cells, served as a useful internal control to exclude any potential variation in fixation and staining. The material included two patients (both TRG4), who received preoperative chemotherapy only and the difference in cancer cell nuclear staining between tumors remained significant following exclusion of these patients (TRG1-3 *vs* TRG4: *p* = 0.001, cf Table 1). There was no significant difference in pERK staining between tumors located at low or middle positions in the rectum (*p* = 0.7; only two patients had tumors positioned higher).

Grading of cancer cell nuclear staining in blind-coded sections stained with the second monoclonal pERK antibody (E10) confirmed a significant difference between TRG1-3 and TRG4 (Mann-Whitney test: *p* = 0.015) and the scores for cancer cell nuclear staining correlated positively with both antibodies (Spearman rho = 0.738, *p* < 0.0001).

**Figure 5:**
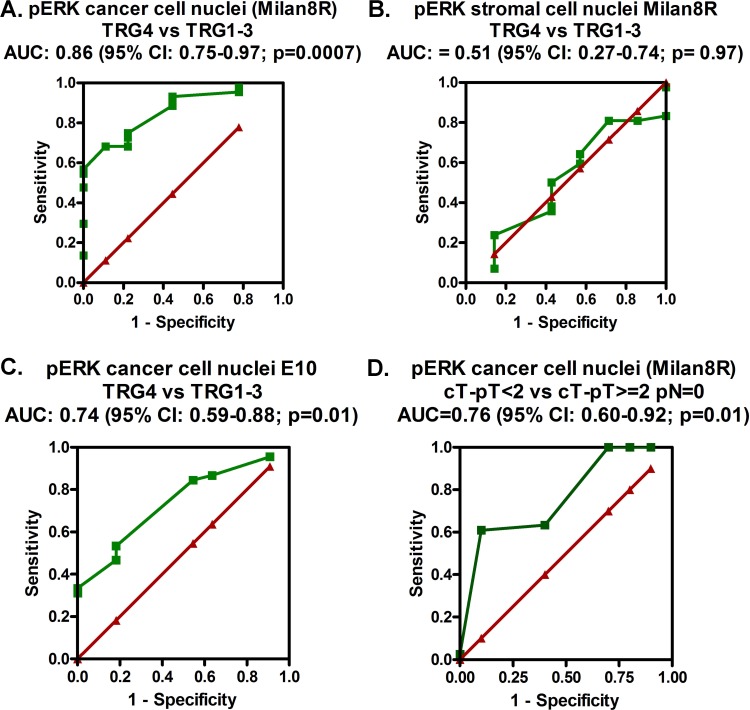
ROC curves (green) demonstrating the prediction accuracy (TRG4 *vs* TRG1-3) of staining of cancer cell nuclei **A.** and stromal cell nuclei **B.** with the pERK (Milan8R) antibody and of staining of cancer cell nuclei **C.** with the pERK (E10) antibody as well as the prediction accuracy for downstaging (defined as being equal to or exceeding a reduction of 2 in the clinical versus pathological T stage with no positive lymph nodes as detected by pathological examination) of staining of cancer cell nuclei with the pERK (Milan8R) antibody **D.** The red lines illustrate imaginary curves, which show no prediction accuracy (AUC = 0.5).

We used receiver operating characteristic (ROC) curves for evaluating the prediction accuracy of the pERK stainings. Cancer cell nuclear staining with the Milan8R pERK antibody potently separated TRG4 from TRG1-3 (AUC: 0.86; 95% C.I. 0.75-0.97) (Figure 5A). Staining of stromal cell nuclei showed no significant separation (Figure 5B). Also cancer cell nuclear staining with the E10 pERK antibody separated TRG4 from TRG1-3 (AUC: 0.74; 95% C.I.: 0.59-0.88) (Figure 5C). Finally, staining for pERK also showed a good predictive power for downstaging (equal to or exceeding a reduction of 2 in the clinical versus pathological T stage with no positive lymph nodes as detected by pathological examination: Milan8R antibody: AUC = 0.76; 95% C.I.: 0.60-0.92) (Figure 5D).

## DISCUSSION

Our results show that use of high pH demasking permits use of higher dilutions of pERK antibodies and that it results in much more intense staining than low pH demasking, which up to now has been used in pERK immunolocalization studies of formalin-fixed, paraffin embedded material. Staining of stromal cells served as a valuable internal positive control and was uniform throughout the sections with no evidence of a gradient. This is important because variations in block size and/or in ischemia time may compromise staining of these highly labile phosphoepitopes [16-18]. The staining of stromal cells in CRC is not unexpected because ERK activation is important both to angiogenesis and fibroblast proliferation [19, 20] - phenomena that characterize most cancers.

Our optimized immunohistochemical protocol demonstrated that staining for pERK in nuclei of cancer, but not stromal, cells correlates to the degree of response to RCT and that higher levels of nuclear pERK are associated with poorer responses. Results with two different monoclonal pERK antibodies were similar. However, staining with the Milan8R antibody was crisper and, although both antibodies predicted the response to RCT, statistics obtained with the Milan8R antibody was best. Moreover, cancer cell nuclear staining for pERK showed a significant predictive power with respect to downstaging. A limitation of our study was that pretreatment biopsies from only 52 patients were available. It will therefore be of interest to perform a confirmatory study on a larger patient material and such a study is now underway. However, once this is said, the statistics obtained in the present study appear very robust. In addition, it would be interesting to examine the effects of RCT on levels of pERK in the surgical resections. However, due to variable delays in time before fixation of surgical resections, referred to above, pERK staining may be compromised. Thus, even an ischemia time of 1h may severely compromise staining results [16-18]. This problem is never encountered in freshly fixed biopsies. For this reason, as well as for our wish to determine whether pERK immunohistochemistry could be used to predict the response to RCT, the present study was restricted to freshly fixed pretreatment biopsies.

Our findings may reflect a radioprotective effect of pERK. Thus, previous studies have documented that interference with ERK phosphorylation increases radiosensitivity of cancer cells in culture [10, 11]. A major effect of radiotherapy is to induce DNA damage and apoptosis in cancer cells. Survivin - an antiapoptotic protein, the expression of which is stimulated by ERK signaling [4, 8-9] may possibly participate in mediating this effect. Interestingly, in some studies, survivin has been shown to be a marker for RCT responses [see 4 and references therein]. It remains an exciting topic for future investigations to see if survivin expression and ERK activation correlate to each other in rectal adenocarcinoma. Moreover, our results make it tempting to speculate whether inhibitors of ERK phosphorylation (MEK inhibitors), which already are in clinical trials, may improve the response rate of rectal carcinomas to RCT.

## MATERIALS AND METHODS

### Biopsy material

Pretreatment biopsies from 52 consecutive patients, selected for RCT during 2006-2013 at the Copenhagen University Hospital at Hvidovre, were immediately fixed in buffered formalin and processed for paraffin embedding. Biopsies from 7 patients that had been examined by private practicing pathologists were, however, not available. Patient data are summarized in the text and in Table 1. Part of this material has been used in a previous study of another marker [21]. Fifty patients received radiotherapy (48-60 Gy over 25-27 days), whereas two patients (TRG4) did not receive radiotherapy. Fifty-one patients received concomitant treatment with 5-fluorouracil (Xeloda), which was terminated after 2 weeks in 3 patients (2 TRG1, 1 TRG4). Additionally, three patients also received concomitant oxaliplatin and one of these also received concomitant avastatin treatment (all TRG3). Pathological tumor staging (pT), nodal metastases (pN), vascular and neural invasion as well as differentiation were assessed on hematoxylin and eosin stained sections of surgical resection specimens. Tumor regression grade (TRG) was evaluated according to Mandard et al. [14, 15]. According to this classification, TRG1 means complete regression and TRG4 implies a minimal response. Tumor location was assessed by visual inspection of resection specimens, which included the anorectal junction, or else by magnetic resonance (MR) imaging data. In addition, clinical tumor stage (cT) was derived from MR data. Such data were available for 46 of the 52 patients (TRG1: 8/9; TRG2: 13/16; TRG3: 17/18; TRG4: 8/9. Major downstaging were defined as cT-pT≥2 pN = 0. The study was approved by the Danish Data Protection Agency (2008-41-2252) and Ethical Committee (H-KF-26288/KF-01-164/03).

### Immunohistochemistry

Three-μm paraffin sections were deparaffinized and stained with 2 different mouse monoclonal antibodies specific to active, dually phosphorylated ERK1/2 (clone Milan8R; mouse IgG1, eBioscience Affymetrix, San Diego, CA and clone E10; mouse IgG1, #9106, Cell Signaling Technology, Danvers, MA). Both monoclonals are specific for activating phosphorylations of Thr202/Tyr204 in ERK1 and of Thr185/Tyr187 in ERK2. Initially, biopsies from six rectal adenocarcinomas were stained in order to titrate the antibodies and to test different demasking procedures. Deparaffinized, hydrated sections were demasked either at high pH (Tris/EDTA buffer, pH 9, using the Dako K8000 kit: Envision^TM^ FLEX target retrieval solution, high pH, Dako, Glostrup, Denmark) or low pH (citrate buffer pH 6.1, using K8005: Envision^TM^ FLEX target retrieval solution, low pH, Dako) in a PT Link machine (Dako). Examination of adjacent sections clearly documented the superiority of high pH demasking (cf Figure 1). The optimized procedure consisted of demasking at high pH, followed by staining with the monoclonal phospho-ERK1/2 Milan8R antibody at 0.125 μg/ml and detection with a two-layer Envision (Dako Envision^TM^ FLEX, high pH (Link) K8000 kit) peroxidase detection system using a Dako autostainer. Additional sections were stained using the E10 monoclonal antibody, followed by the Envision K8000 detection system. Sections were counterstained with hematoxylin. Controls included type-matched control mouse IgG1 directed to Aspergillus niger glucose oxidase, which is absent from mammalian tissues (clone DAK-GO1, Dako, Glostrup, Denmark), at the same concentration as the phospho-ERK1/2 Milan8R monoclonal antibody. In addition, other sections underwent high pH demasking, as described above, and were then dephosphorylated using 4800 units lambda protein phosphatase (New England Biolabs, Ipswich, MA) in 0.6 ml NEB buffer (50 mM HEPES, 100 mM NaCl, 2 mM DTT, 0.01 % Brij 35 pH 7.5, supplemented with 1 mM MnCl_2_) for 60 min at 30°C prior to immunohistochemical staining.

### Scoring and statistics

Immunohistochemically stained sections were blind-coded and, following an initial survey, staining intensities of tumor and stromal cell nuclei were graded as nil (0), moderate (1) or strong (2) and numbers of stained cancer and stromal cell nuclei were graded as 0% (0), below 10% (1), 10-60% (2) and above 60 % (3). The intensity scores were multiplied with the number scores before statistical analyses. Specimens were scored by two independent observers (SH and LIL), who were blinded for the clinical outcome. Comparisons between scores yielded a kappa value of 0.74. Final results were calculated as an average of the original scores of both observers. Statistical analysis (non-parametric) was performed using SAS (version 9.3, SAS Institute, Cary, NC) and GraphPad Prism (version 4, GraphPad Software, Inc., La Jolla, CA). The significance level was set at 5%.
